# Supplementary material for: Mechanism of DNA unwinding by MCM8-9 in complex with HROB
Source: Nat Commun. 2024 Apr 27;15:3584. doi: 10.1038/s41467-024-47936-8 (PMC11055865; doi:10.1038/s41467-024-47936-8)
Supplement: Supplementary file 1 — Supplementary Information [file 41467_2024_47936_MOESM1_ESM.pdf]

Supplementary Information

Mechanism of DNA unwinding by MCM8-9 in complex with HROB

Ananya Acharya[1,2], Hélène Bret[3], Jen-Wei Huang[4], Martin Mütze[5], Martin Göse[5], Vera Maria Kissling[2], Ralf Seidel[5], Alberto Ciccia[4], Raphaël Guérois[3] and Petr Cejka[1,2]

Affiliations:

[1]Institute for Research in Biomedicine, Università della Svizzera italiana (USI), Faculty of Biomedical Sciences, Bellinzona, 6500, Switzerland

[2]Department of Biology, Institute of Biochemistry, Eidgenössische Technische Hochschule (ETH), Zürich, 8093, Switzerland

[3]Université Paris-Saclay, CEA, CNRS, Institute for Integrative Biology of the Cell (I2BC), 91198, Gif-sur-Yvette, France.

[4]Department of Genetics and Development, Institute for Cancer Genetics, Herbert Irving Comprehensive Cancer Center, Columbia University Irving Medical Center, New York, NY, USA

[5]Peter Debye Institute for Soft Matter Physics, Universität Leipzig, Leipzig, 04103, Germany.

Vera Maria Kissling - present address: Particles-Biology Interactions Laboratory, Department of Materials Meet Life, Swiss Federal Laboratories for Materials Science and Technology (Empa), St. Gallen, 9014, Switzerland

Correspondence:
Petr Cejka; petr.cejka@irb.usi.ch
Raphaël Guérois; raphael.guerois@cea.fr

Inventory

Supplementary Figures 1-11

Supplementary Figure Legends

Supplementary Tables S1 to S4

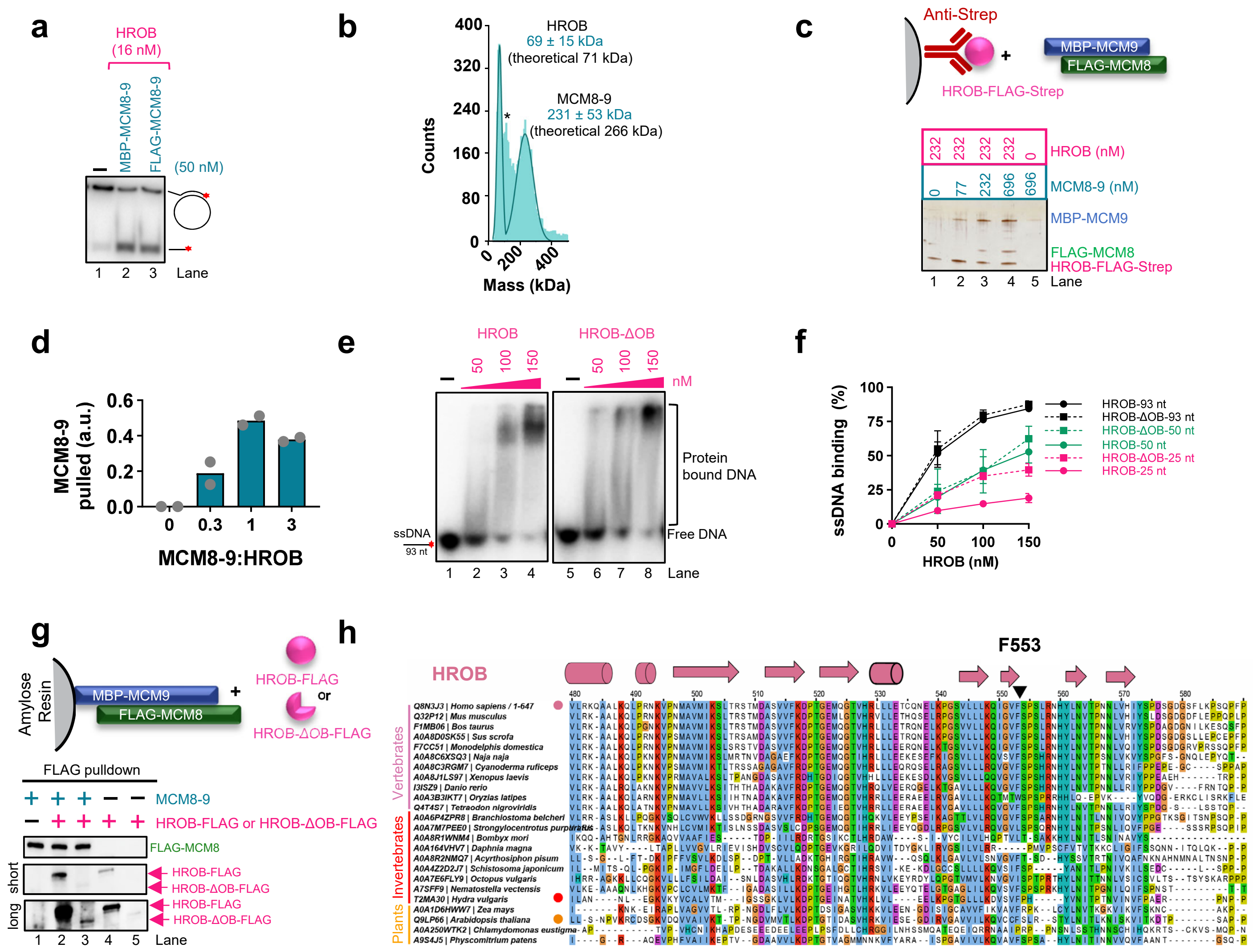

Supplementary Fig. 1. Interaction of HROB with MCM8-9.

a. A comparison of DNA unwinding by FLAG-MCM9 and untagged MCM8, or MBP-MCM9 and FLAG-MCM8, purified as a complex, in the presence of HROB using circular M13-based ssDNA. The red asterisk indicates the position of the radioactive label. The MBP and FLAG tags were not cleaved from the corresponding proteins. Shown is a representative of n = 3 independent experiments.
b. Measured molecular weight distribution of the interaction intermediate of MCM8-9 and HROB at low concentrations (100 nM) using mass photometry. Error, SD. FLAG-MCM8-MBP-MCM9 and HROB-FLAG were used, corresponding to a theoretical molecular weight of 266 kDa and 71 kDa respectively. No combined complex made up of all the proteins (theoretical molecular weight 337 kDa) was observed. * represents background noise or truncations of the protein preparation.
c. Physical interaction assay of HROB-FLAG-Strep (232 nM) (bait) with 0.3 (77 nM), 1x (232 nM) and 3x (696 nM) stoichiometric amounts of MCM8-9 (prey). The Strep-tagged HROB was immobilized on anti-Strep-tag II antibody. MCM8-9 was subsequently added, bound proteins were eluted, and subjected to silver staining. Top, a schematic of the assay; bottom, a representative silver stained gel of n = 2 independent experiments.
d. Quantification of assays such as shown in panel c; data points show range; n = 2.
e. Electrophoretic mobility shift assay with human HROB or HROB-ΔOB and 93 nt-long ssDNA. The red asterisk indicates the position of the radioactive label. Shown is a representative of n = 3 independent experiments.
f. Quantification of electrophoretic mobility shift assay with human HROB or HROB-ΔOB and 25, 50 and 93 nt-long ssDNA (dashed: HROB-ΔOB, solid: HROB). Error bars, SEM; n = 3 independent experiments. The values for 93 nt ssDNA are replotted from Fig. 1c for reference.
g. MBP-tagged MCM8-9 from *Sf*9 cell lysate was immobilized on amylose resin (bait) and incubated with HROB variants (141 nM) (prey) at 100 mM NaCl. Bound proteins were eluted and visualized by western blotting with anti-FLAG antibody, which detected FLAG-tagged MCM8 and FLAG-tagged HROB. Top, a schematic of the assay; bottom, representative western blots of n = 2 independent experiments. The lowest blot shows a higher exposure of the blot above.
h. Multiple sequence alignment of HROB showing evolutionary conservation of F553 and the surrounding region.

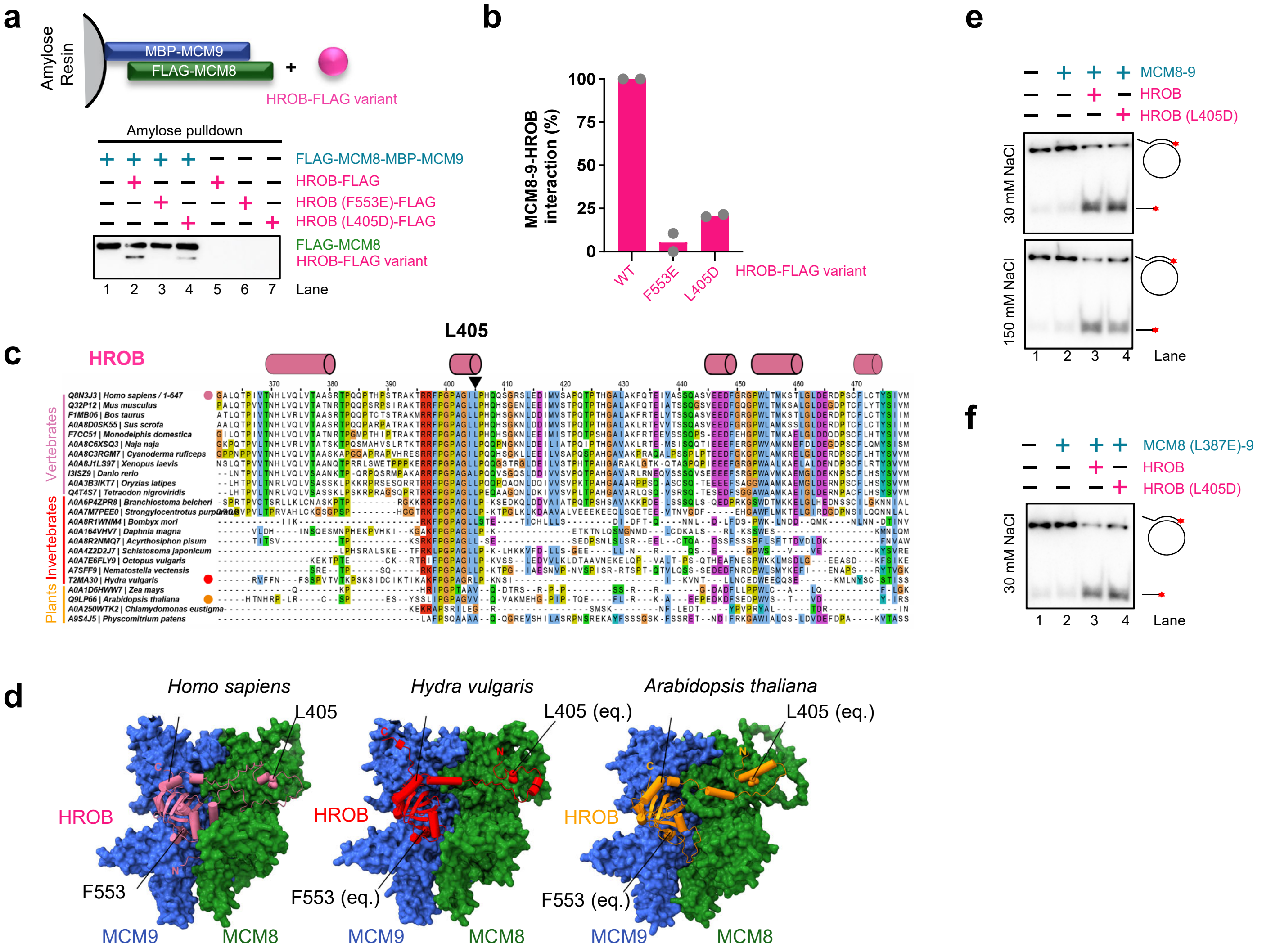

Supplementary Fig. 2. HROB interacts with MCM8-9 via both MCM8 and MCM9 subunits.

a. MBP-tagged MCM8-9 from *Sf*9 cell lysate was immobilized on amylose resin (bait) and incubated with witd type HROB or variants (141 nM) (prey) at 100 mM NaCl. Bound proteins were eluted and visualized by western blotting with anti-FLAG antibody, which detected FLAG-tagged MCM8 and FLAG-tagged HROB. Top, a schematic of the assay; bottom, representative western blots of n = 2 independent experiments.
b. Quantification of assays such as shown in panel a; data points show range; n = 2 independent experiments. The values were normalized to wild type HROB (lane 2).
c. Multiple sequence alignment of HROB showing the conservation of L405 and the surrounding region.
d. A cartoon showing a conservation of the interaction of HROB with MCM8-9 in various species, modelled by AlphaFold2.
e. DNA unwinding with HROB (L405D) (25 nM) and MCM8-9 (50 nM) to test for the impact of disrupting HROB interaction with MCM8, carried out at indicated NaCl concentrations using circular M13-based DNA. The red asterisk indicates the position of the radioactive label. Shown is a representative of n = 3 independent experiments.
f. Assay as panel e, except using MCM8 (L387E)-9 variant. Shown is a representative of n = 3 independent experiments.

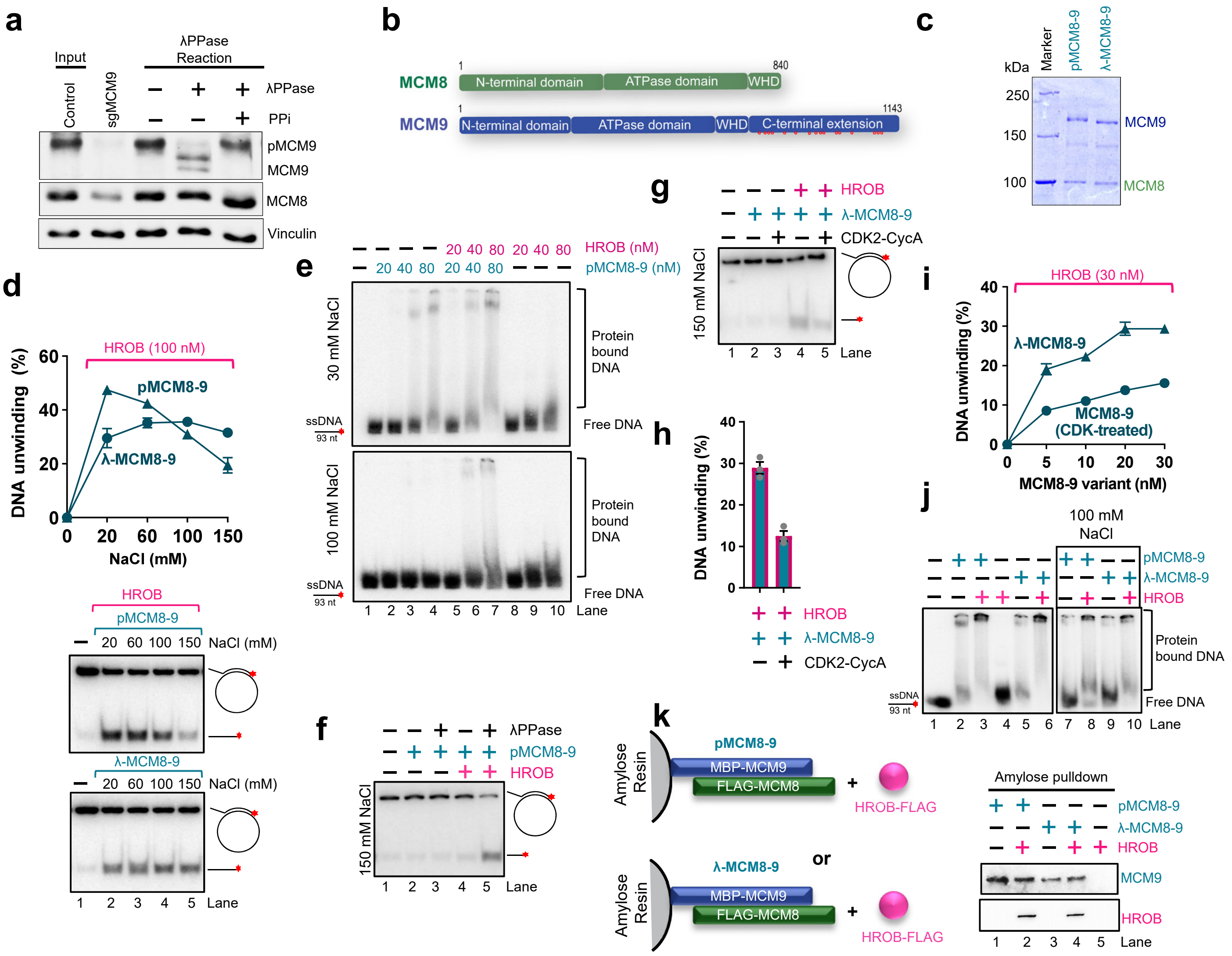

Supplementary Fig. 3. Regulation of MCM8-9 by phosphorylation and DNA binding and unwinding specificity of MCM8-9.

a. Endogenous MCM9 is phosphorylated. Lysates from HEK293T control cells (input) were treated with lambda phosphatase (λPPase) in the absence or presence of phosphatase inhibitors (PPi) and resolved by SDS-PAGE supplemented with Phos-Bind. Immunoblotting of MCM9 and MCM8 is presented along with a loading control, vinculin. Specificity of the MCM9 band is demonstrated by the loss of signal in lysates from cells stably transduced with LentiCRISPRv2-Puro sgMCM9. Shown is a representative of two independent experiments.

b. A schematic representation of the main domains in human MCM8 and MCM9. WHD, winged-helix domain. The red dots represent approximate positions of 16 putative consensus CDK phosphorylation sites present in the unique C terminal tail of MCM9 (T673, S703, S707, S711, S762, S802, T879, S883, S890, S915, S942, S952, T977, T1064, S1073 and S1088).

c. MCM8-9 heterodimer variants prepared with phosphatase inhibitors (phosphorylated MCM8-9, pMCM8-9), or without phosphatase inhibitors and treated with λ phosphatase during purification (dephosphorylated MCM8-9, λ-MCM8-9). The phosphorylated form of MCM9 shows a mobility shift in gel electrophoresis. Shown is a representative of two independent preparations.

d. DNA unwinding assays to compare the activities of phosphorylated (pMCM8-9) and dephosphorylated (λ-MCM8-9) MCM8-9 variants (both 100 nM), with HROB (100 nM), and its dependence on NaCl concentration using circular M13-based DNA. The red asterisk indicates the position of the radioactive label. Top, quantification; error bars, SEM; n = 3; bottom, a representative experiment.

e. Electrophoretic mobility shift assay with pMCM8-9 in the presence or absence of HROB using a 93 nt long ssDNA with 30 and 100 mM NaCl and 3 mM EDTA. The red asterisk indicates the position of the radioactive label. Panel shows representative experiments of n = 3.

f. DNA unwinding by phosphorylated human pMCM8-9 (100 nM) with or without lambda phosphatase (λPPase) treatment, with or without HROB (30 nM) using M13-based circular DNA substrate. The red asterisk indicates the position of the radioactive label. Shown is a representative experiment.

g. DNA unwinding by de-phosphorylated human MCM8-9 (100 nM), treated or not with CDK2-CycA, with or without HROB (30 nM) at 150 mM NaCl using M13-based circular DNA substrate with 150 mM NaCl. The red asterisk indicates the position of the radioactive label. Shown is a representative experiment.

h. Quantification of assays such as shown in panel g. Error bars, SEM; n = 3.

i. Quantification of DNA unwinding experiments by human MCM8-9, either phosphorylated with model CDK, CDK2-CycA, MCM8-9 (CDK), or dephosphorylated, λ-MCM8-9, at 150 mM NaCl using circular M13-based DNA. Error bars, SEM; n = 3.

j. Electrophoretic mobility shift assays with pMCM8-9 or λ-MCM8-9 (both 100 nM), in the presence or absence of HROB (100 nM) with or without added salt (100 mM NaCl) and 93 nt-long ssDNA with 3 mM EDTA. Panel shows a representative experiment n = 3.

k. Physical interaction assay with pMCM8-9 (bait) or λ-MCM8-9 (bait) and HROB (prey) at 100 mM NaCl. The MBP-tagged MCM8-9 variants from *Sf*9 cell extracts were immobilized on amylose resin, HROB (141 nM) was subsequently added, bound proteins were eluted, and subjected to western blotting. MCM9 was detected with anti-MCM9 and HROB with anti-FLAG antibodies. Left, a schematic of the assay and right, representative blots of n = 2.

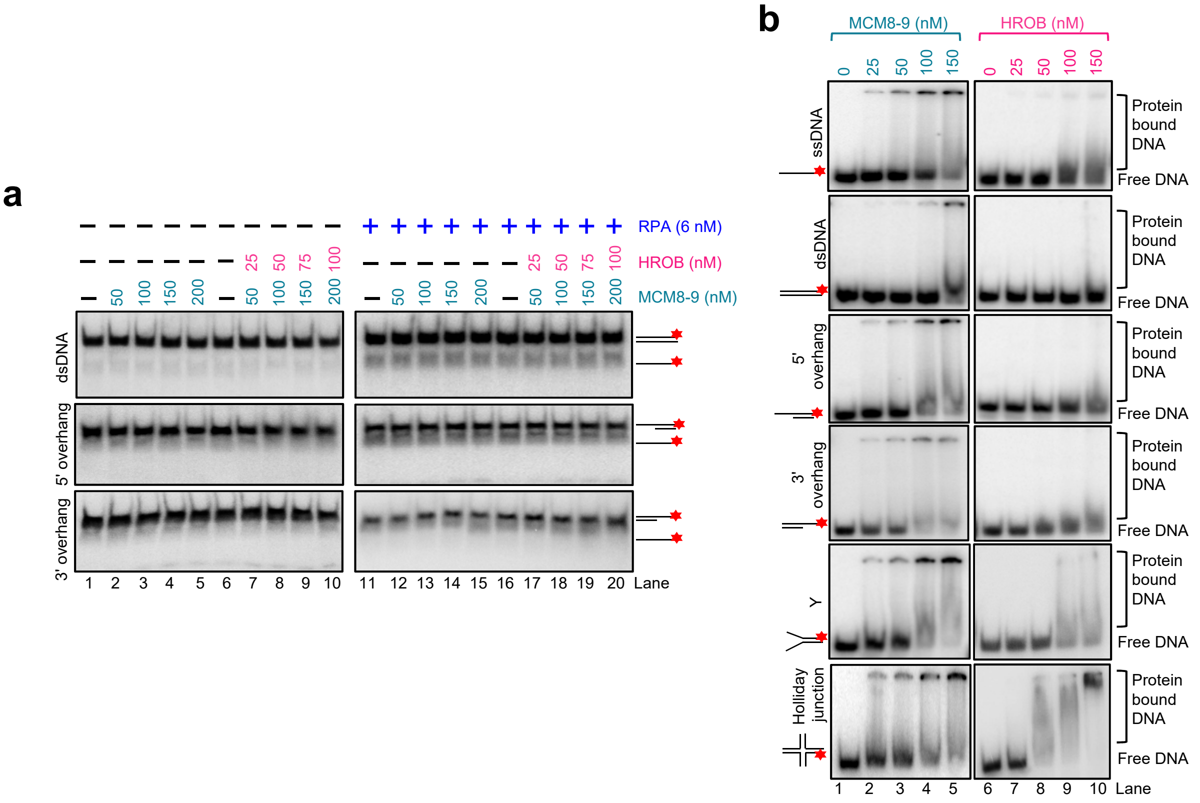

Supplementary Fig. 4. DNA substrate specificity of MCM8-9 and HROB.

a. DNA unwinding by pMCM8-9 without or with HROB and RPA, using oligonucleotide-based DNA substrates with 1 mM ATP, 5 mM magnesium acetate and 15 mM NaCl. Representatives of n = 3 independent experiments are shown. The red asterisk indicates the position of the radioactive label.

b. Electrophoretic mobility shift assays with pMCM8-9 or HROB, using various oligonucleotide-based DNA substrates with 3 mM EDTA. Representatives of n = 3 independent experiments are shown. The red asterisk indicates the position of the radioactive label.

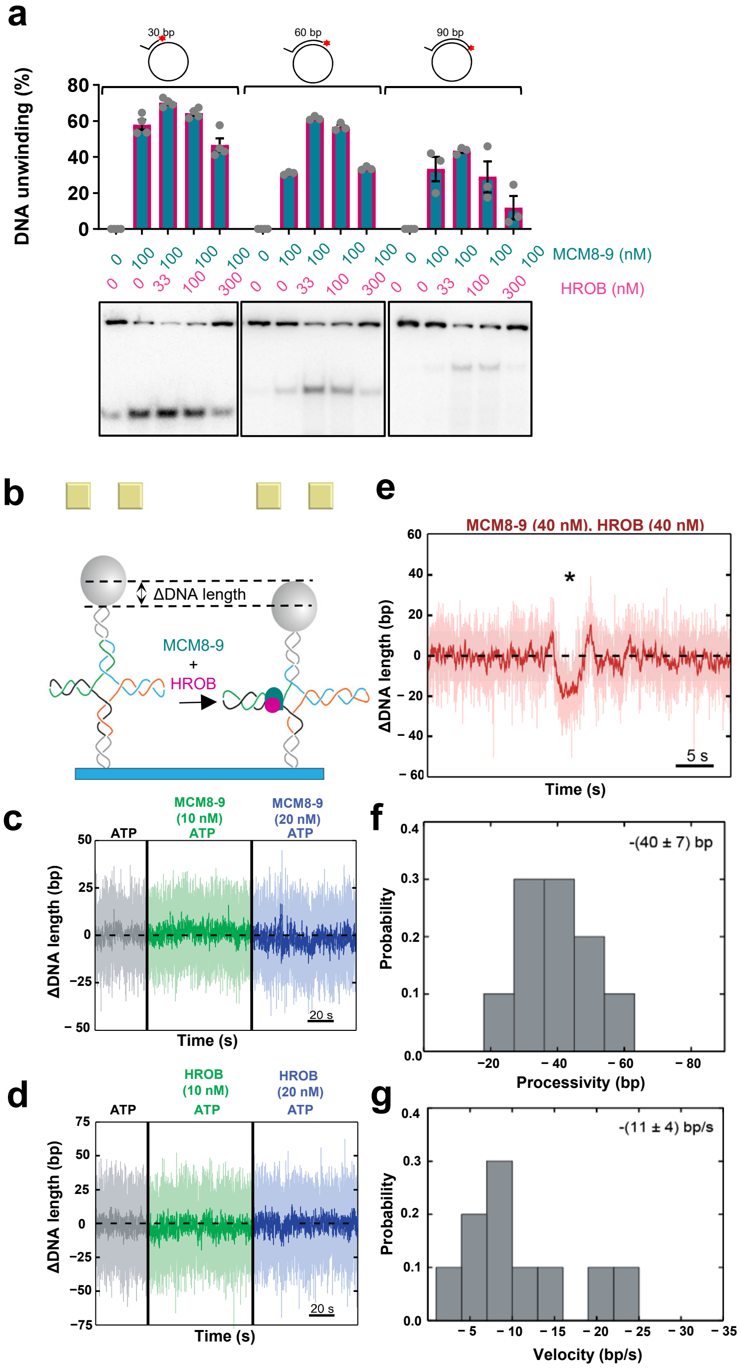

Supplementary Fig. 5. Magnetic tweezer experiments with the MCM8-9 helicase and HROB.

a. DNA unwinding by pMCM8-9 without and with various amounts of HROB, using M13-based substrates with 5' overhang (substrates contained 30, 60 and 90 bp of duplex DNA, overhang length 20 nt). Top, quantification; representatives for data points in lanes 1-5 n = 4 independent experiments; for data points in lanes 6-15 n = 3 independent experiments are shown; error bars, SEM; bottom.

b. A schematic representation of the magnetic tweezer setup and the investigated Holliday junction construct with 262 bp in each arm. When the protein ensemble is added, MCM8-9 with the cofactor HROB can translocate in the vertical direction, causing the arms of the Holliday junction to extend further, leading to a net-decrease in DNA length.

c. Processing of the Holliday junction construct by MCM8-9 alone without HROB. No protein activity was detected.

d. Processing of the Holliday junction construct by HROB alone. No protein activity was detected.

e. An example trace of a net-downward event (highlighted by *) at a concentration of 40 nM for MCM8-9:HROB (1:1).

f. Probability distribution of DNA unwinding processivity by MCM8-9 with HROB, with a mean of –(40 ± 7) bp, of events leading to DNA shortening. DNA unwinding of 10 molecules was measured.

g. Probability distribution of DNA unwinding velocity by MCM8-9 with HROB, with a mean of –(11 ± 4) bp sec^{-1}, of events leading to DNA shortening. DNA unwinding of 10 molecules was measured.

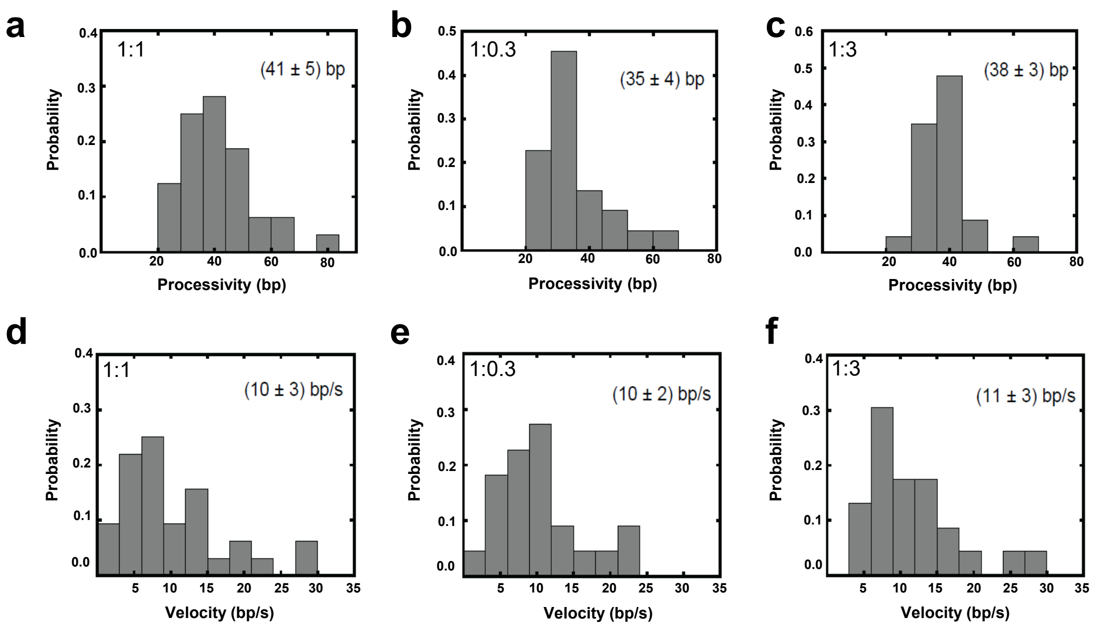

Supplementary Fig. 6. Magnetic tweezer experiments show that the ratio of MCM8-9 helicase and HROB does not alter the DNA unwinding speed and processivity.

a-c. Probability distributions of positive unwinding processivity for the concentration ratios of 1:1, 1:0.3 and 1:3 of MCM8-9 with HROB. The mean values are 41 ± 5 bp for the 1:1 ratio, 35 ± 4 bp for the 1:0.3, and 38 ± 3 bp for the 1:3 ratio. The 1:1 ratio is replotted as in Fig. 3f for comparison. DNA unwinding was measured for 22 and 23 molecules in panel b and c respectively.

d-f. Probability distributions of positive unwinding velocity for the concentration ratios of 1:1, 1:0.3 and 1:3 of MCM8-9 with HROB. The mean values are 10 ± 3 bp sec^{-1} for the 1:1 ratio, 10 ± 2 bp sec^{-1} for the 1:0.3, and 11 ± 3 bp sec^{-1} for the 1:3 ratio. The 1:1 ratio is replotted as in Fig. 3g for comparison. DNA unwinding was measured for 22 and 23 molecules in panel e and f respectively.

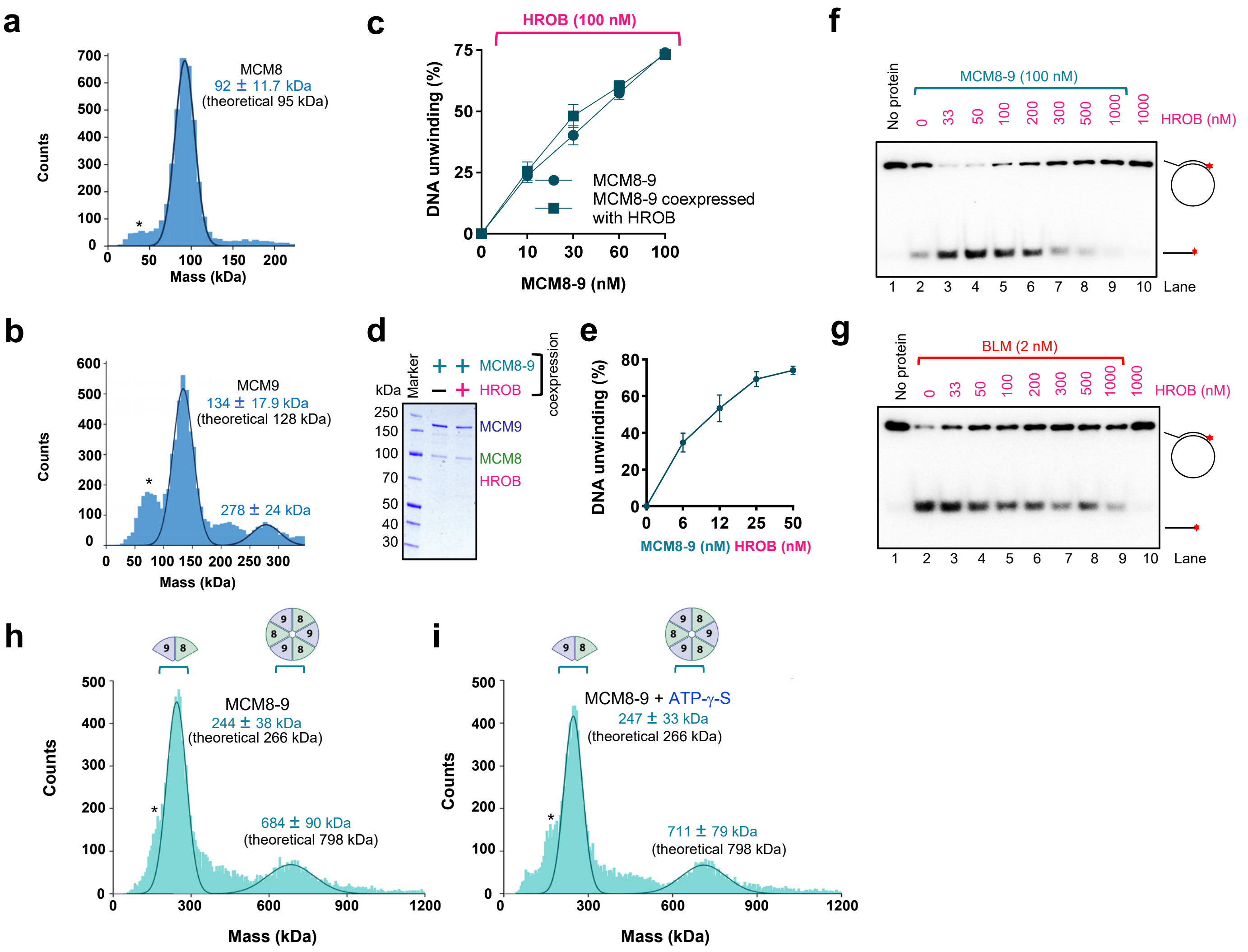

Supplementary Fig. 7. Analysis of MCM8-9 oligomerization.

a. Measured molecular weight distribution of human FLAG-MCM8 (theoretical value 95 kDa including tag) using mass photometry. Error, SD. * represents background noise or truncations in the protein preparation. MCM8 was FLAG-tagged.

b. Measured molecular weight distribution of human MCM9-FLAG (theoretical value 128 kDa including tag) using mass photometry. Error, SD. * represents background noise or truncations in the protein preparation. MCM9 was FLAG-tagged.

c. Quantification of helicase assays with MCM8-9 complex expressed as a heterodimer or co-expressed with HROB. MCM8-9 was 100 nM. As MCM8-9 did not co-purify with HROB, all reactions were supplemented with 100 nM HROB. Error bars, SEM; n = 3 independent experiments.

d. Purified recombinant MCM8-9 complex expressed together with HROB, as used in panel c. HROB could not be co-purified with MCM8-9. Shown is a representative of n = 2 independent gels.

e. Quantification of DNA unwinding assays using equimolar concentrations of MCM8-9 and HROB, as indicated, using M13-based circular DNA substrate. Error bars, SEM; n = 3 independent experiments .

f. Representative DNA unwinding assays using human pMCM8-9 in the presence of various HROB concentrations using M13-based circular DNA substrate at 32 mM NaCl. Representative of n = 3 independent experiments is shown.

g. Representative DNA unwinding assays with human BLM in the presence of various HROB concentrations using M13-based circular DNA substrate at 32 mM NaCl. Representative of n = 3 independent experiments is shown.

h. Measured molecular weight distribution of human MCM8-9 (theoretical value 266 kDa for the heterodimer, 798 kDa for the hexamer, including tags) using mass photometry. Errors, SD. * represents background noise or truncations in the protein preparation. MCM8 was FLAG-tagged and MCM9 was MBP-tagged.

i. Experiment as in panel h, but in a buffer additionally containing 2 mM ATP-γ-S and 1 mM magnesium acetate.

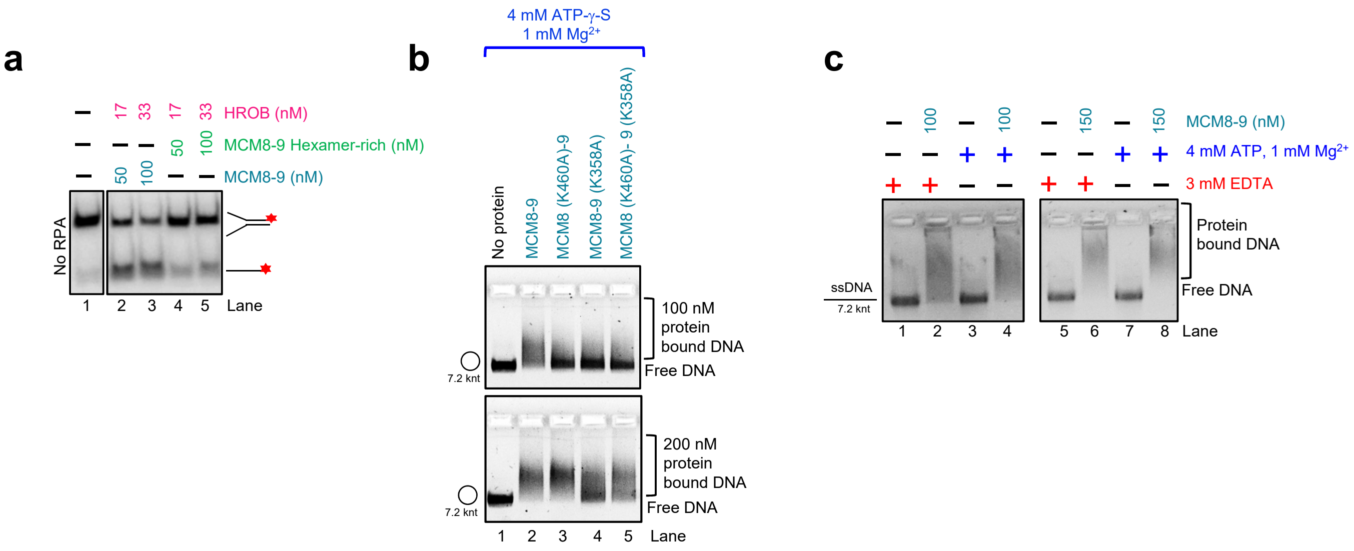

Supplementary Fig. 8. DNA binding and unwinding by MCM8-9.

a. Representative DNA unwinding assays of n = 3 independent experiments with standard and hexamer-rich MCM8-9 preparations in the presence HROB, using Y-structured DNA substrate. The red asterisk indicates the position of the radioactive label.

b. Electrophoretic mobility shift assays with human MCM8-9, with ATP-γ-S, as indicated, using M13-based circular ssDNA substrate. Representative of n = 3 independent experiments is shown.

c. Electrophoretic mobility shift assays with human MCM8-9 without or with ATP, using M13-based linear ssDNA (7.2 knt). A representative of n = 3 independent experiments is shown.

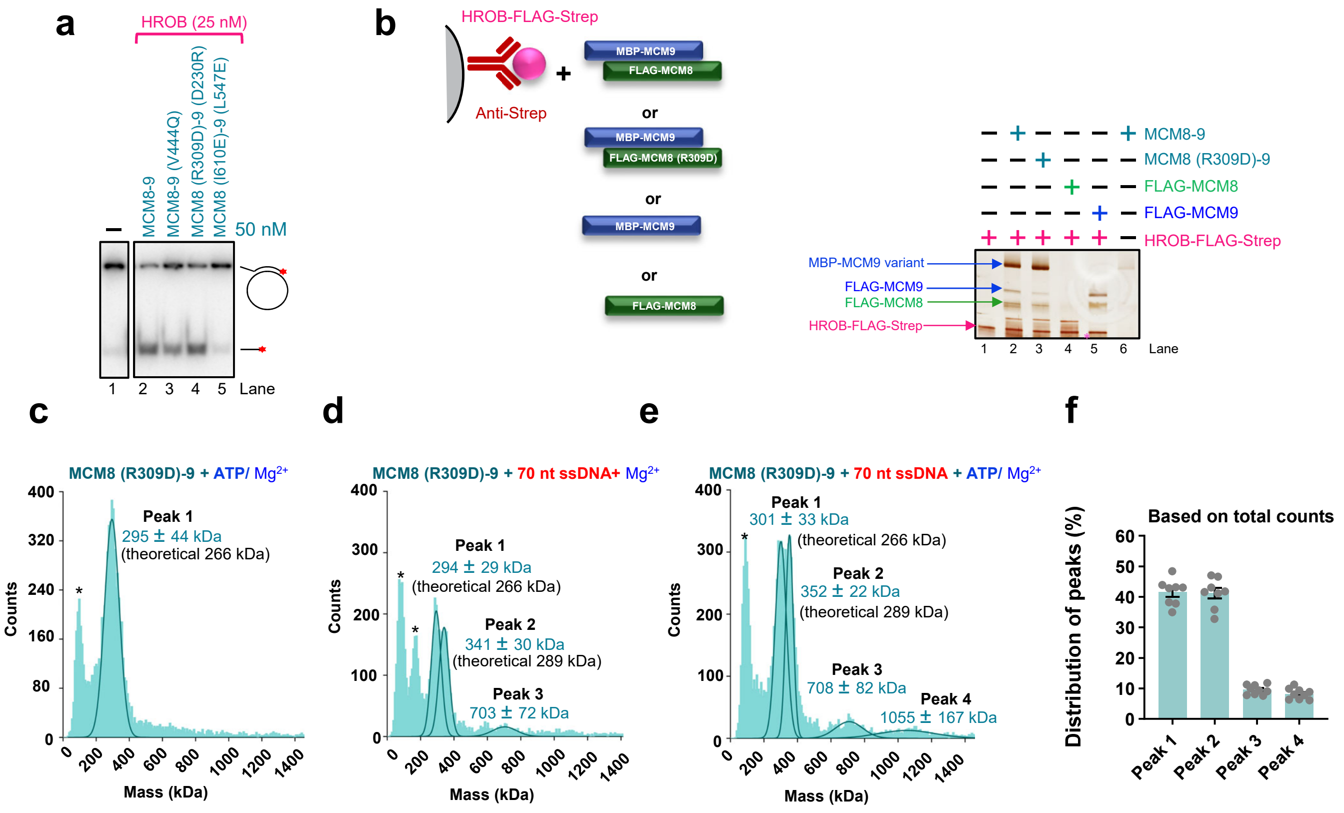

Supplementary Fig. 9. Analysis of MCM8-9 mutants disrupting the N-terminal part of interface II.

a. Representative DNA unwinding assays of n = 3 independent experiments with MCM8-9 variants (50 nM) mutated in interface II, as indicated, at 25 mM NaCl in the presence of HROB (25 nM) using M13-based circular DNA substrate.

b. Physical interaction assay of HROB-FLAG-Strep (232 nM, 1 µg) (bait) with equimolar amounts of MCM8-9 wild type (mixture of dimers and hexamers), MCM8-9 dimer mutant (MCM8 (R309D)-9), MCM8 monomer and MCM9 monomer (preys). The MCM8-9 complex variants were purified as FLAG-MCM8-MBP-MCM9 and the monomers were purified as FLAG-MCM8 and FLAG-MCM9. The Strep-tagged HROB was immobilized using the anti-strep-tag II antibody. Prey proteins were subsequently added, bound proteins were eluted, and subjected to silver staining. Left, a schematic of the assay; right, a representative silver stained gel of n = 2 independent experiments.

c-e. Mass photometry measurements showing molecular weight distribution of MCM8 (R309D)-MCM9 mutant with 70-nt long ssDNA and/or 2 mM ATP and 1 mM magnesium acetate, as indicated. Errors, SD. * represents background noise, DNA or protein truncations. MCM8 was FLAG-tagged and MCM9 was MBP-tagged. (c) MCM8 (R309D)-MCM9 was monitored with ATP/Mg^{2+}, corresponding to a theoretical molecular weight of 266 kDa for the heterodimer. No defined higher molecular weight complexes were observed. MCM8 (R309D)-MCM9 was monitored with DNA (d), but without ATP/Mg^{2+}. The theoretical molecular weight of the 70 nt ssDNA is 23 kDa. Peak 1 is free MCM8 (R309D)-MCM9, Peak 2 is MCM8 (R309D)-MCM9 bound to DNA. Not well-defined Peak 3 likely corresponds to two MCM8-9 heterodimers bound independently to DNA. (e) MCM8 (R309D)-MCM9 was monitored with DNA and ATP/Mg^{2+}. Peak 1 is free MCM8 (R309D)-MCM9, Peak 2 is MCM8 (R309D)-MCM9 bound to DNA. Peak 3 represents two MCM8 (R309D)-MCM9 heterodimers and Peak 4 is likely the hexameric complex bound to DNA. ATP and DNA enhanced the proportion of species corresponding to the hexamer (Peak 4).

f. Distribution of peaks obtained in the experiments such as shown in panel (e), based on the total number of counts. Error, SEM; 8 molecules were counted for Peak 1 and 2 and 9 molecules were counted for for Peak 3 and 4. Peak 3 likely corresponds to two MCM8 (R309D)-MCM9 heterodimers bound to DNA independently by chance, as it is much less represented than Peak 2, corresponding to one heterodimer bound to DNA. If Peak 4 corresponded to three heterodimers bound to DNA independently by chance, it would be much less frequent than Peak 3, which is not the case. Peak 4 thus likely represents the formation of a functional hexameric complex on DNA in the presence of ATP/Mg^{2+}. Although the fraction of peak 4 appears to be small (~ 10%), each species in this peak has 3x the molecular weight of species from Peak 1. If we correct for the molecular weight, the fraction of total protein mass present in Peak 4 is almost as high as in Peak 1 (~ 30%).

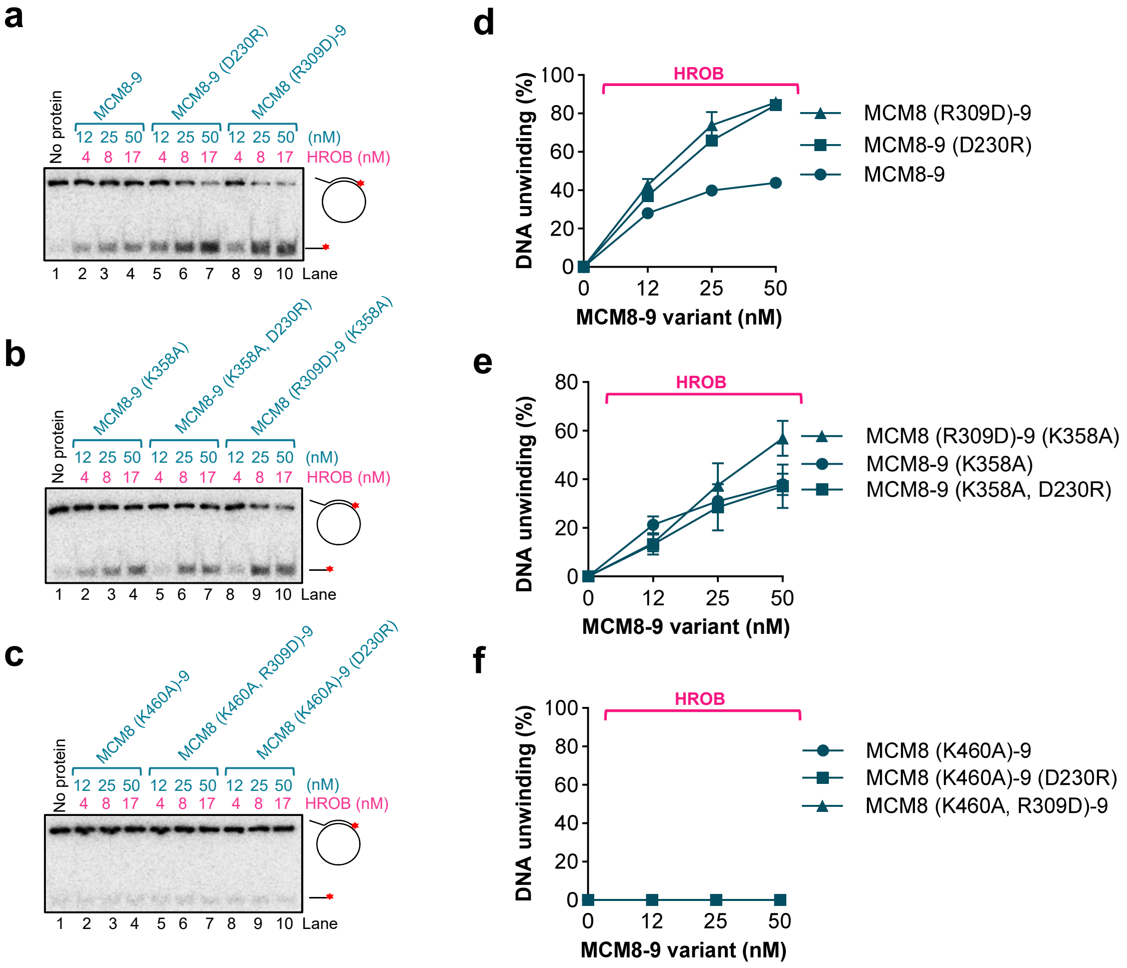

Supplementary Fig. 10. Analysis of MCM8-9 mutations disrupting the N-terminal part of interface II.

a-c. DNA unwinding by MCM8-9 variants with disrupted N-terminal part of interface II, in combination with ATPase Walker A mutations, as indicated, at 25 mM NaCl, in the presence of HROB, using M13-based circular DNA substrate. Panels show representative experiments of n = 3 independent experiments.

d-f. Quantification of assays such as shown in panels a-c. HROB was used at 1/3 of the indicated concentration of MCM8-9 (nM). Error bars, SEM; n = 3 independent experiments.

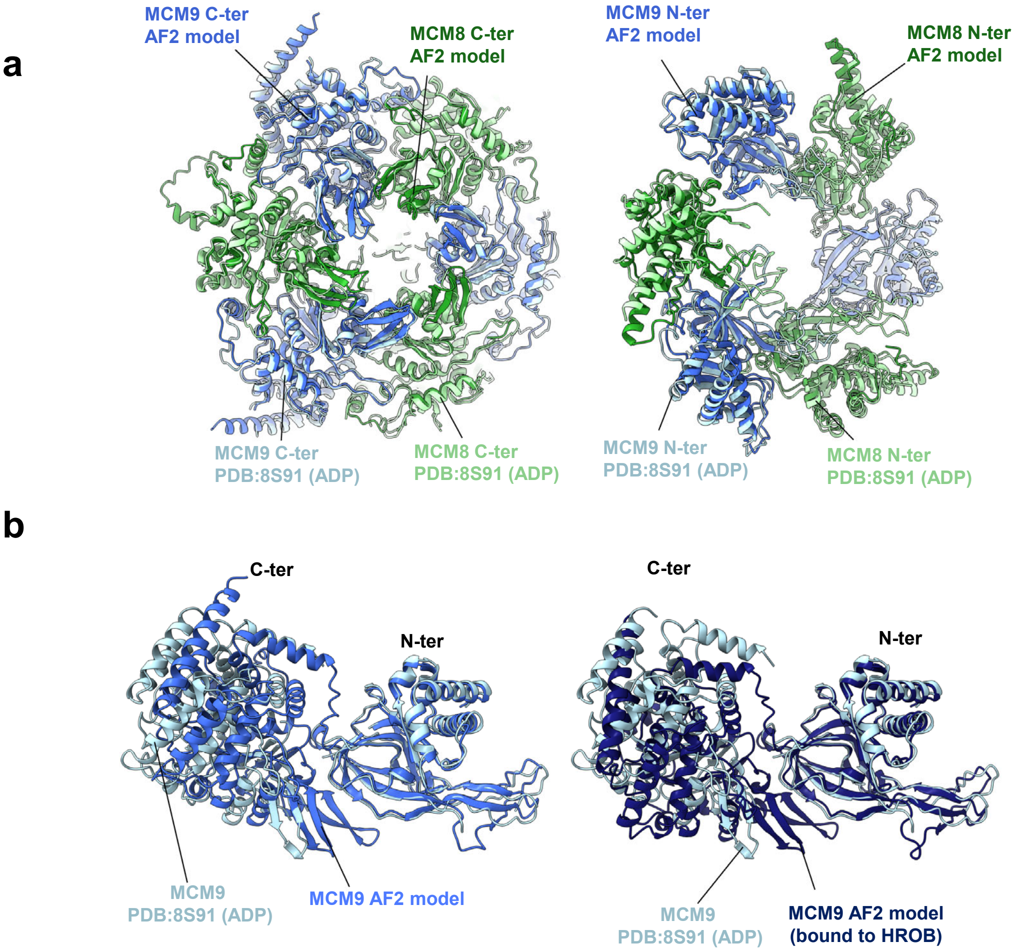

Supplementary Fig. 11. Comparison of AlphaFold2 model of MCM8-9 with experimental structure (PDB: 8S91, Ref[20]).

a. Comparison of the MCM8-9 C-terminal ring modeled by AlphaFold2 and experimental structure PDB: 8S91 (left, RMSD 1.9 Å), and MCM8-9 N-terminal ring modeled by AlphaFold2 with PDB: 8S91 (right, RMSD 2.2 Å).

b. Comparison of the relative orientations of MCM9 N-terminal and C-terminal domains modeled by AlphaFold2 and experimental structure PDB: 8S91. Left, AlphaFold2 model of MCM9, extracted from the MCM8-9 hexamer modeled without HROB. Right, AlphaFold2 model of MCM9, extracted from the MCM8-9 hexamer modeled with HROB.

Table S1: Protein sequence of HROB-FLAG used in this study.

**Protein**	**Protein Sequence**
**HROB-FLAG**	MACSLQKLFAVEEEFEDEDFLSAVEDAENRFTGSLPVNAGRLRPVSSRPQETVQAQSSRL<br>LLLHPTAPSEALGLPDLDLCLPASSTPSADSRPSCIGAAPLRPVSTSSSWIGNQRRVTVTEV<br>LREPARPQSSALHPLLTFESQQQQVGGFEGPEQDEFDKVLASMELEEPGMELECGVSSEA<br>IPILPAQQREGSVLAKKARVVDLSGSCQKGPVPAIHKAGIMSAQDESLDPVIQCRTPRPPL<br>RPGAVGHLPVPTALTVPTQQLHWEVCPQRSPVQALQPLQAARGTIQSSPQNRFPCQPFQS<br>PSSWLSGKAHLPRPRTPNSSCSTPSRTSSGLFPRIPLQPQAPVSSIGSPVGTPKGPQGALQTP<br>IVTNHLVQLVTAASRTPQQPTHPSTRAKTRRFPGPAGILPHQQSGRSLEDIMVSAPQTPTH<br>GALAKFQTEIVASSQASVEEDFGRGPWLTMKSTLGLDERDPSCFLCTYSIVMVLRKAALK<br>QLPRNKVPNMAVMIKSLTRSTMDASVVFKDPTGEMQGTVHRLLLETCQNELKPGSVLLL<br>KQIGVFSPSLRNHYLNVTPNNLVHIYSPDSGDGSFLKPSQPFPKDSGSFQHDVAAKPEEGF<br>RTAQNLEAEASPEEELPEADDLDGLLSELPEDFFCGTSSGGTSDYKDDDDK

Table S2: List of oligonucleotides used for site-directed mutagenesis to produce recombinant proteins

Primer	Sequence (5'-3')
HROB-F553E Forward Primer	CTGAAGCAGATTGGAGTGGAGTCTCCTTCACTTCGAAATCAC
HROB-F553E Reverse Primer	GTGATTTCGAAGTGAAGGAGACTCCACTCCAATCTGCTTCAG
HROB-L405D Forward Primer	CCCTGGCCCAGCTGGGATCGACCCTCACCAGCAGAGTG
HROB-L405D Reverse Primer	CACTCTGCTGGTGAGGGTCGATCCCAGCTGGGCCAGGG
MCM8 R309D Forward Primer	CGTGAGGCTGGTCGTATCCCTGACACCATCGAGTGCGAGCTG
MCM8 R309D Reverse Primer	CAGCTCGCACTCGATGGTGTCAGGGATACGACCAGCCTCACG
MCM8 I610E Forward Primer	CTCCTGTCCGAGCACGTGGAGGCTATCCGTGCTGGTAAG
MCM8 I610E Reverse Primer	CTTACCAGCACGGATAGCCTCCACGTGCTCGGACAGGAG
MCM8 L387E Forward Primer	GGTATGCTGATGGAGTTCTCCGAGAAGGACCTGTACGCTATC
MCM8 L387E Reverse Primer	GATAGCGTACAGGTCCTTCTCGGAGAACTCCATCAGCATACC
MCM8 E314R Forward Primer	CCTCGTACCATCGAGTGCCGGCTGGTGCACGACTTGGTG
MCM8 E314R Reverse Primer	CACCAAGTCGTGCACCAGCCGGCACTCGATGGTACGAGG
MCM8 V547Q Forward Primer	CTGGCTAAGGCTGGTGTGCAGTGCTCCCTGCCTGCTCGC
MCM8 V547Q Reverse Primer	GCGAGCAGGCAGGGAGCACTGCACACCAGCCTTAGCCAG
MCM8 R697D Forward Primer	GACTTCTACCTGGAGCTGGACAAGCAGTCCCAGCGTCTG
MCM8 R697D Reverse Primer	CAGACGCTGGGACTGCTTGTCCAGCTCCAGGTAGAAGTC
MCM9 V444Q Forward Primer	GTGGCCAAAGCAGGACTGCAGTGCAAACTAAACACGCGAACC
MCM9 V444Q Reverse Primer	GGTTCGCGTGTTTAGTTTGCACTGCAGTCCTGCTTTGGCCAC
MCM9 L547E Forward Primer	GGGAACCAAGTACTTGAGCGCTACTATCAGATG
MCM9 L547E Reverse Primer	CATCTGATAGTAGCGCTCAAGTACTTGGTTCCC
MCM9 R256E Forward Primer	CCCTTTCAACAAGATGTAGAGTGTGAAGTGGAGATTGTG
MCM9 R256E Reverse Primer	CACAATCTCCACTTCACACTCTACATCTTGTTGAAAGGG
MCM9 D230R Forward Primer	CTGGAAGATGATCTGGTGCGCTCCTGCAAGAGTGGTGATG
MCM9 D230R Reverse Primer	CATCACCACTCTTGCAGGAGCGCACCAGATCATCTTCCAG
MCM9 L506E Forward Primer	GAATAATAAGCTCCTTCATCGAGGAGAACAAAGGGTATCCCTCC
MCM9 L506E Reverse Primer	GGAGGGATACCCTTTGTTCTCCTCGATGAAGGAGCTTATTATTC
MCM9 M45E Forward Primer	CCAGTGGTGGTAAATGCCGAGACGCTCTTTGAAACCAAC
MCM9 M45E Reverse Primer	GTTGGTTTCAAAGAGCGTCTCGGCATTTACCACCACTGG
MCM8 K460A Forward Primer	TTGGAGATCCAGGCCTAGGAGCTAGTCAAATGCTACAGGCAGC
MCM8 K460A Reverse Primer	GCTGCCTGTAGCATTTGACTAGCTCCTAGGCCTGGATCTCCAA
MCM9 K358A Forward Primer	TTGGGGATCCTGGCACAGGGGCTTCTCAGTTCCTCAAATATGC
MCM9 K358A Reverse Primer	GCATATTTGAGGAACTGAGAAGCCCCTGTGCCAGGATCCCCAA
MCM8 L387E Forward Primer	GGAGTTCTCAGAGAAAGACCTTTATGCC
MCM8 L387E Reverse Primer	ATCAACATTCCATGCTTAC
MCM9 M45E Forward Primer	GGTTAATGCCGAGACTCTGTTTGAGAC
MCM9 M45E Reverse Primer	ACAACTGGGTAATGAGCATC

Table S3: Sequence of oligonucleotides used for substrate preparation in this study.

Name	Sequence (5'-3')
X12-3 HJ3	GAGATCTATCTGGTGCCTTCTGACAGTGAATGGGTAACGAATC GTAATAGTCTCTAGACAGCATGTCCTAGCAATGTAATCGTCTA TGACGTC
M13-5'-dT 40 overhang	TTTTTTTTTTTTTTTTTTTTTTTTTTTTTTTTTTTTTTTTGTTTTCC CAGTCACGACGTTGTAAAACGACGGCCAGT
M13-37mer no overhang	GTTTTCCCAGTCACGACGTTGTAAAACGACGGCCAGT
M13-3'-dT 40 overhang	GTTTTCCCAGTCACGACGTTGTAAAACGACGGCCAGTTTTTTT TTTTTTTTTTTTTTTTTTTTTTTTTTTTTTTTT
M13-5'-dT20overhang-30bp_c	TTTTTTTTTTTTTTTTTTTTGTTTTCCCAGTCACGACGTTGTAAA ACGAC
M13 5' dT20overhang-60bp_c	TTTTTTTTTTTTTTTTTTTTGTTTTCCCAGTCACGACGTTGTAAA ACGACGGCCAGTGCCAAGCTTGCATGCCTGCAGGT
M13 5' dT20overhang-90bp_c	TTTTTTTTTTTTTTTTTTTTGTTTTCCCAGTCACGACGTTGTAAA ACGACGGCCAGTGCCAAGCTTGCATGCCTGCAGGTCGACTCTA GAGGATCCCCGGGTACCGAGCT
M13 5' dT5overhang-30bp_c	TTTTTGTTTTCCCAGTCACGACGTTGTAAAACGAC
M13 3' dT5overhang-30bp_c	GTTTTCCCAGTCACGACGTTGTAAAACGACTTTTT
M13-overhang-30bp_c	GTTTTCCCAGTCACGACGTTGTAAAACGAC
PC1253	TGGGTCAACGTGGGCAAAGATGTCCTAGCAATGTAATCGTCTA TGACGTT
PC1254	TGCCGAATTCTACCAGTGCCAGTGATGGACATCTTTGCCCACG TTGA CCC
PC1255	GTCGGATCCTCTAGACAGCTCCATGATCACTGGCACTGGTAGA ATTC GGC
PC1256	CAACGTCATAGACGATTACATTGCTACATGGAGCTGTCTAGAG GATC CGA
PC1253C	AACGTCATAGACGATTACATTGCTAGGACATCTTTGCCCACGT TGAC CCA
312	AACGTCATAGACGATTACATTGCTA
314	CATGGAGCTGTCTAGAGGATCCGAC
PC216	GTAAGTGCCGCGGTGCGGGTGCCAGGGCGTGCCCTTGGGCTC CCCGGGCGCGTACTCCACCTCATGCATC
HJ-fragment 4229 bp Forward Primer	GGCGCCGCCTCAGCCGCAGGAAAGAACATGTGA
HJ-fragment 4229 bp Reverse Primer	TTATCGATACCGTCGACCTCGAGCAGCAACCGCA
HJ-fragment 5326 bp Forward Primer	GGCGCCGCCTCAGCCGCAGGAAAGAACATGTGA
HJ-fragment 5326 bp Reverse Primer	AAACAGGCTCTGCGGGCCCGGCGT
Handles Forward Primer	GACCGAGATAGGGTTGAGTG
Handles Reverse Primer	TTTGTGATGCTCGTCAGGGG

Table S4: List of oligonucleotides used for site-directed mutagenesis to produce plasmids for cellular assays in this study.

Primer	Sequence (5'-3')
MCM8 L387E Forward Primer	GGAGTTCTCAGAGAAAGACCTTTATGCC
MCM8 L387E Reverse Primer	ATCAACATTCCATGCTTAC
MCM9 M45E Forward Primer	GGTTAATGCCGAGACTCTGTTTGAGAC
MCM9 M45E Reverse Primer	ACAACTGGGTAATGAGCATC
