# Supplementary material for: Mechanism of DNA unwinding by MCM8-9 in complex with HROB
Source: Nat Commun. 2024 Apr 27;15:3584. doi: 10.1038/s41467-024-47936-8 (PMC11055865; doi:10.1038/s41467-024-47936-8)
Supplement: Supplementary file 3 — Reporting Summary [file 41467_2024_47936_MOESM3_ESM.pdf]

nature portfolio

Corresponding author(s): Petr Cejka
Raphael Guerois

Last updated by author(s): Mar 28, 2024

Reporting Summary

Nature Portfolio wishes to improve the reproducibility of the work that we publish. This form provides structure for consistency and transparency in reporting. For further information on Nature Portfolio policies, see our Editorial Policies and the Editorial Policy Checklist.

Statistics

For all statistical analyses, confirm that the following items are present in the figure legend, table legend, main text, or Methods section.

n/a	Confirmed	
☐	☒	The exact sample size (*n*) for each experimental group/condition, given as a discrete number and unit of measurement
☐	☒	A statement on whether measurements were taken from distinct samples or whether the same sample was measured repeatedly
☒	☐	The statistical test(s) used AND whether they are one- or two-sided<br>*Only common tests should be described solely by name; describe more complex techniques in the Methods section.*
☒	☐	A description of all covariates tested
☒	☐	A description of any assumptions or corrections, such as tests of normality and adjustment for multiple comparisons
☐	☒	A full description of the statistical parameters including central tendency (e.g. means) or other basic estimates (e.g. regression coefficient) AND variation (e.g. standard deviation) or associated estimates of uncertainty (e.g. confidence intervals)
☒	☐	For null hypothesis testing, the test statistic (e.g. *F*, *t*, *r*) with confidence intervals, effect sizes, degrees of freedom and *P* value noted<br>*Give P values as exact values whenever suitable.*
☒	☐	For Bayesian analysis, information on the choice of priors and Markov chain Monte Carlo settings
☒	☐	For hierarchical and complex designs, identification of the appropriate level for tests and full reporting of outcomes
☒	☐	Estimates of effect sizes (e.g. Cohen's *d*, Pearson's *r*), indicating how they were calculated

Our web collection on statistics for biologists contains articles on many of the points above.

Software and code

Policy information about availability of computer code

Data collection	Gels were acquired using Typhoon Phosphor lmager FLA 9500, version 1.0, Quantum CX5 Edge 18.06 and photo scanner operated with CanoScan 9000F Mark II scanner and lmageCapture v6.6(525) software. Blots were captured using Fusion FX7 Edge 18.12-5. Magnetic tweezers single molecule data were collected using Labview 2016 and CUDA software as published (Huhle et al, Nat Commun, 2015). AcquireMP (Refeyn Ltd, version 2023 Rl.l) was used for mass photometry data collection.
Data analysis	The radioactive gels in this study were analyzed using lmageJ 1.53g and the results were plotted using Graph Pad Prism 10.l.0 (316). Data analysis as well as plotting of magnetic tweezers data was carried out in Origin Lab 2019. DiscoverMP (Refeyn Ltd, version v2023 Rl.2) was used for mass photometry data analysis. Structural models for each of the HROB-MCM8-MCM9 trimer and of the MCM8-MCM9 hexamer were generated using a local version of the ColabFold vl.5.2 interface running iterations of the Alphafold2 v2.3.l algorithm trained on the multi mer dataset on a local HPC equipped with NVIDIA Ampere Al00 80Go GPU cards.

For manuscripts utilizing custom algorithms or software that are central to the research but not yet described in published literature, software must be made available to editors and reviewers. We strongly encourage code deposition in a community repository (e.g. GitHub). See the Nature Portfolio guidelines for submitting code & software for further information.

Data

Policy information about availability of data

All manuscripts must include a data availability statement. This statement should provide the following information, where applicable:

- Accession codes, unique identifiers, or web links for publicly available datasets
- A description of any restrictions on data availability
- For clinical datasets or third party data, please ensure that the statement adheres to our policy

The structural models are available in ModelArchive (modelarchive.org) with the accession codes ma-ji514 and ma-ma-zlpye for the MCM8-MCM9 hexamer and HROB-MCM8-MCM9 complex, respectively. Mass photometry movies and magnetic tweezer data are uploaded to Dryad [DOI: 10.5061/dryad.wdbrv15wq]. The previously published experimental structure of human ADP-bound MCM8-9 8S91 [DOI: 0.2210/pdb8S91/pdb] was used as a reference. Uncropped gel images and numerical source data for graphs are included in the source data file. All easily replicable material (plasmids) are available from the corresponding author upon reasonable request.

Research involving human participants, their data, or biological material

Policy information about studies with human participants or human data. See also policy information about sex, gender (identity/presentation), and sexual orientation and race, ethnicity and racism.

Reporting on sex and gender	N/A
Reporting on race, ethnicity, or other socially relevant groupings	N/A
Population characteristics	N/A
Recruitment	N/A
Ethics oversight	N/A

Note that full information on the approval of the study protocol must also be provided in the manuscript.

Field-specific reporting

Please select the one below that is the best fit for your research. If you are not sure, read the appropriate sections before making your selection.

☒ Life sciences ☐ Behavioural & social sciences ☐ Ecological, evolutionary & environmental sciences

For a reference copy of the document with all sections, see nature.com/documents/nr-reporting-summary-flat.pdf

Life sciences study design

All studies must disclose on these points even when the disclosure is negative.

Sample size	Sample size (or number of repeats) was chosen based on what is common or is practical to do in the field (Huang et al, 2020, Acharya et al, 2021, Kissling et al, 2022).
Data exclusions	In general, no data were excluded unless there was a valid reason to do so, e.g. experiments with failed positive controls indicating technical problems, or when loading control indicated unequal loading that invalidated the analysis or other technical issues (broken gels, collapsed wells in gels etc.).
Replication	The experiments were replicated several times as indicated in figure legends. Oftentimes, even within a single experiment, several protein concentrations were analyzed, or samples were compared at multiple time points. This also contributes to the reliability of the data.
Randomization	Randomization is not relevant to the experiments performed in this study. Our readouts are exactly quantifiable (fraction of DNA unwinding, amount of protein pulled down, etc.). Therefore, there is no risk of introducing a bias.
Blinding	Blinding is not relevant to the experiments in this study, as measurements were objectively quantified by dedicated software or simply visually presented. Furthermore, the loading order of samples on gels prevented blinding.

Reporting for specific materials, systems and methods

We require information from authors about some types of materials, experimental systems and methods used in many studies. Here, indicate whether each material, system or method listed is relevant to your study. If you are not sure if a list item applies to your research, read the appropriate section before selecting a response.

Materials & experimental systems

n/a	Involved in the study	
☐	☒	Antibodies
☐	☒	Eukaryotic cell lines
☒	☐	Palaeontology and archaeology
☒	☐	Animals and other organisms
☒	☐	Clinical data
☒	☐	Dual use research of concern
☒	☐	Plants

Methods

n/a	Involved in the study	
☒	☐	ChIP-seq
☒	☐	Flow cytometry
☒	☐	MRI-based neuroimaging

Antibodies

Antibodies used	rabbit anti-HROB (Sigma HPA023393; 1:1,000 dilution), rabbit anti-MCM8 (Proteintech 16451-1-AP; 1:5000 dilution), mouse anti-HA (Sigma H3663; 1:2,500 dilution), mouse anti-vinculin (Sigma V9131; 1:20,000 dilution) rabbit anti-MCM9 (Millipore ABE2603; 1:10,000 dilution), mouse anti-FLAG (Sigma F1804; 1:1,000 dilution) and mouse anti-Strep-tag II antibody (Abcam, ab184224)
Validation	The antibodies used in this study are available commercially with specificity and application data available online. Applications in our study for each antibody are listed in the manuscript. In particular, the specificities of HROB, MCM8 and MCM9 antibodies were validated with some combination of sgRNA-mediated deletion, siRNA-induced depletion, and/or cDNA (wildtype and mutant) overexpression studies in cell lines by western blotting and/or immunoprecipitation previously reported (see Huang et al, 2020, PMID: 32528060).

Eukaryotic cell lines

Policy information about cell lines and Sex and Gender in Research

Cell line source(s)	We used Sf9 cells adapted for suspension growth, available from the cell line collection of the Institute of Molecular Cancer Research, University of Zurich. HEK293T were obtained from ATCC.
Authentication	The cell line was not authenticated.
Mycoplasma contamination	The cell line was not tested for mycoplasma contamination.
Commonly misidentified lines (See ICLAC register)	No misidentified cell lines were used.

Plants

Seed stocks	*Report on the source of all seed stocks or other plant material used. If applicable, state the seed stock centre and catalogue number. If plant specimens were collected from the field, describe the collection location, date and sampling procedures.*
Novel plant genotypes	*Describe the methods by which all novel plant genotypes were produced. This includes those generated by transgenic approaches, gene editing, chemical/radiation-based mutagenesis and hybridization. For transgenic lines, describe the transformation method, the number of independent lines analyzed and the generation upon which experiments were performed. For gene-edited lines, describe the editor used, the endogenous sequence targeted for editing, the targeting guide RNA sequence (if applicable) and how the editor was applied.*
Authentication	*Describe any authentication procedures for each seed stock used or novel genotype generated. Describe any experiments used to assess the effect of a mutation and, where applicable, how potential secondary effects (e.g. second site T-DNA insertions, mosiacism, off-target gene editing) were examined.*
